# Analysis of the Element-Free Galerkin Method with Penalty for Stokes Problems

**DOI:** 10.3390/e24081072

**Published:** 2022-08-03

**Authors:** Tao Zhang, Xiaolin Li

**Affiliations:** 1 School of Mathematics, Physics and Data Science, Chongqing University of Science & Technology, Chongqing 401331, China; zhangtao895701777@126.com; 2 School of Mathematical Sciences, Chongqing Normal University, Chongqing 400047, China

**Keywords:** meshless methods, element-free Galerkin method, Stokes problems, error estimate

## Abstract

The element-free Galerkin (EFG) method with penalty for Stokes problems is proposed and analyzed in this work. A priori error estimates of the penalty method, which is used to deal with Dirichlet boundary conditions, are derived to illustrate its validity in a continuous sense. Based on a feasible assumption, it is proved that there is a unique weak solution in the modified weak form of penalized Stokes problems. Then, the error bounds with the penalty factor for the EFG discretization are derived, which provide a rationale for choosing an efficient penalty factor. Numerical examples are given to confirm the theoretical results.

## 1. Introduction

It is well-known that Stokes equations describe low Reynolds number flow motion and play a fundamental role in the numerical modeling of incompressible viscous flows. Recently, there has been an increasing interest in solving Stokes problems by various meshfree (or meshless) methods [1,2,3,4,5] to alleviate mesh-related dilemmas, including some collocation meshless methods, such as virtual interpolation point method [6], generalized finite difference method [7], divergence-free kernel approximation method [8], as well as some Galerkin meshless methods, such as the moving least square reproducing kernel method [9], weighted extended B-spline method [10], Galerkin boundary node method [11], and the divergence-free meshless local Petrov–Galerkin method [12].

The element-free Galerkin (EFG) method [13] is a Galerkin-based meshfree discretization technique for solving partial differential equations. The trial and test functions for the EFG method are generated by the moving least squares (MLS) approximation [14]. During the past several decades, many research works have been devoted to improving and extending the MLS approximation, see [4,15,16,17,18] for various details. To offset the lack of interpolating properties of the MLS shape functions, several interpolation-type MLS methods have been developed. We refer to [14,18,19,20,21] and the references therein for details.

In addition to choosing the interpolation-type MLS methods, some mandatory methods, such as the Lagrange multiplier method [3,4,13], Nitsche method [22,23] and penalty method [3,4,24,25,26,27,28] are desirable in practical applications. The important feature of these methods is that they can straightforwardly use the non-interpolating trial and test functions by modifying the traditional weak form. The penalty method seems to be more appealing because of its ease of implementation, its variable-preservation and its general framework, and these significant advantages enable numerical analysis.

In the context of the EFG method, many papers are devoted to penalty-based error analysis for elliptic problems [24,25], parabolic problems [26,28] and contact problems [27]. To the authors’ knowledge, for Stokes problems, a priori errors of the meshless penalty method have not been explained, and numerical analysis with a penalty factor has not been presented either. The main difficulty may be that the modified weak form based on the penalty method is different from the standard weak form, thus the standard meshless Galerkin procedure cannot be used directly.

In order to better clarify the principle of the penalty method and determine an efficient penalty factor, the presented paper is an extension of the previous works [25,28] on the EFG method for Stokes problems. The modified weak form of penalized Stokes problems is analyzed. Based on a discrete inf-sup condition, error bounds with a penalty factor of the EFG discretization are given in $H^{1}$ norm for velocity approximation and in $L^{2}$ norm for pressure approximation, respectively. Furthermore, an error estimate with a penalty factor for velocity approximation in $L^{2}$ norm is also derived. Numerical examples are given to confirm the theoretical results.

The remainder of the paper is organized as follows. In Section 2, we introduce the Stokes problem and its standard weak formulation. Then, a priori estimates of the penalty method are determined on the Dirichlet boundary and in the problem domain in Section 3, respectively. Section 4 and Section 5 present the modified weak form of the penalized Stokes problem and the EFG numerical discretization, respectively. Section 6 is devoted to the error analysis for velocity and pressure approximations. Numerical examples are presented in Section 7 and conclusions are drawn in the final Section.

Throughout this paper, the letter *C*, with a superscript or subscript, is used to represent a generic positive constant, independent of the characteristic distance *h* and could take different values at different appearances.

## 2. Stokes Problem

Consider the following 2D Stokes problem:

(1) $$\begin{array}{c} \left\{\begin{array}{cc} -\nu \Delta u+\nabla p=f, & \mathrm{in}\;\Omega ,\\ \nabla \cdot u=0, & \mathrm{in}\;\Omega ,\\ u=0, & \mathrm{on}\;\Gamma , \end{array}\right. \end{array}$$

with the velocity $u={\left(u_{1},u_{2}\right)}^{T}$, the pressure *p*, the body force $f={\left(f_{1},f_{2}\right)}^{T}$, and the viscosity ν>0. Assume that the Ω is convex domain, a priori estimate holds [29], i.e.,

(2) $${∥u∥}_{H^{2}\left(\Omega \right)}+{∥p∥}_{H^{1}\left(\Omega \right)}\leq C{∥f∥}_{L^{2}\left(\Omega \right)}.$$

Set $X={\left(H_{0}^{1}\left(\Omega \right)\right)}^{2}$ and $M=L_{0}^{2}\left(\Omega \right)=\left\{q\in L^{2}\left(\Omega \right):\int_{\Omega }qdx=0\right\}$. The weak formulation of (1) is to seek $\left(u,p\right)\in X\times M$ such that

(3) $$A\left(\left(u,p\right);\left(v,q\right)\right)=F\left(v\right),$$

in which $A\left(\left(u,p\right);\left(v,q\right)\right)=a\left(u,v\right)+b\left(v,p\right)+b\left(u,q\right)$ and

$$a\left(u,v\right)=\nu \int_{\Omega }\nabla u\nabla vdx,\;b\left(v,p\right)=-\int_{\Omega }p\nabla \cdot vdx,\;F\left(v\right)=\int_{\Omega }fvdx.$$

Clearly, the bilinear form $a\left(\cdot ,\cdot \right)$ is continuous and coercive on X×X, $b\left(\cdot ,\cdot \right)$ is continuous on X×M and satisfies the inf-sup condition,

(4) $$\underset{v\in X,v\neq 0}{\sup}\frac{\left|b\left(v,q\right)\right|}{\;{∥v∥}_{H^{1}\left(\Omega \right)}}\geq \beta {∥q∥}_{L^{2}\left(\Omega \right)},\;\forall q\in M,$$

where β is a positive constant depending only on Ω. Therefore, the continuity and coercivity of $A\left(\left(\cdot ,\cdot \right);\left(\cdot ,\cdot \right)\right)$ hold, namely

$$\left|A\left(\left(u,p\right);\left(v,q\right)\right)\right|\leq C_{1}\left({∥u∥}_{H^{1}\left(\Omega \right)}+{∥p∥}_{L^{2}\left(\Omega \right)}\right)\left({∥v∥}_{H^{1}\left(\Omega \right)}+{∥q∥}_{L^{2}\left(\Omega \right)}\right),$$

$$\underset{\left(v,q\right)\in X\times M,\left(v,q\right)\neq \left(0,0\right)}{\sup}\frac{\left|A\left(\left(u,p\right);\left(v,q\right)\right)\right|}{{∥v∥}_{H^{1}\left(\Omega \right)}+{∥q∥}_{L^{2}\left(\Omega \right)}}\geq C_{2}\left({∥u∥}_{H^{1}\left(\Omega \right)}+{∥p∥}_{L^{2}\left(\Omega \right)}\right).$$

Then a unique weak solution $\left(u,p\right)\in X\times M$ of (3) follows from the Lax–Milgram theorem.

## 3. Penalized Stokes Problem

In the subsequent numerical discrete process, since the MLS shape functions with non-interpolating properties will be adopted, the penalty method is used to impose the Dirichlet boundary condition. In order to better illustrate the principle of the penalty method, by using a penalty factor α, (1) is approximated as the following penalized problem:

(5) $$\begin{array}{c} \left\{\begin{array}{cc} -\nu \Delta u_{\alpha }+\nabla p=f, & \mathrm{in}\;\Omega ,\\ \nabla \cdot u_{\alpha }=0, & \mathrm{in}\;\Omega ,\\ \nu \frac{\partial u_{\alpha }}{\partial n}-pn+\alpha \nu u_{\alpha }=0, & \mathrm{on}\;\Gamma , \end{array}\right. \end{array}$$

where n is the unit outward normal to Γ. By combining (1) and (5), an a priori estimate of the penalty method on the boundary Γ is first derived.

**Lemma** **1.** *Assume that (5) satisfies the following regularity,*

(6) $${∥u_{\alpha }∥}_{H^{2}\left(\Omega \right)}+{∥p∥}_{H^{1}\left(\Omega \right)}\leq C{∥f∥}_{L^{2}\left(\Omega \right)}.$$

*Then,*

(7) $${∥u_{\alpha }∥}_{L^{2}\left(\Gamma \right)}\leq \frac{C}{\alpha }.$$

**Proof.**  Combining the boundary condition of (5) and the trace theorem [30], we have:

$$\alpha \nu {∥u_{\alpha }∥}_{L^{2}\left(\Gamma \right)}^{2}\leq {∥pn-\nu \frac{\partial u_{\alpha }}{\partial n}∥}_{L^{2}\left(\Gamma \right)}{∥u_{\alpha }∥}_{L^{2}\left(\Gamma \right)}\leq C\left({∥p∥}_{H^{1}\left(\Omega \right)}+{∥u_{\alpha }∥}_{H^{2}\left(\Omega \right)}\right){∥u_{\alpha }∥}_{L^{2}\left(\Gamma \right)},$$

which together with (6) implies (7). □

It is shown by Lemma 1 that when the penalty factor α approaches infinity, the solution $u_{\alpha }$ of (5) tends to 0 with $L^{2}$ norm on the Dirichlet boundary Γ. Clearly, the boundary term pn is almost independent of the penalty factor on this point. In addition, $-\nu \Delta u_{\alpha }+\nabla p=f$ is equivalent to $-\nu \Delta u_{\alpha }=f-\nabla p$, which can be regarded as the approximation of −νΔu=f−∇p. Then, the a priori estimate within the problem domain is exported.

**Lemma** **2.** *Assume that the domain *Ω* is convex and (5) has the regularity condition (6). Then,*

$${∥\nabla \left(u-u_{\alpha }\right)∥}_{L^{2}\left(\Omega \right)}\leq \frac{C}{\sqrt{\alpha }}.$$

**Proof.**  Subtracting (1) from (5) yields:

$$-\nu \Delta \left(u-u_{\alpha }\right)=0.$$

Multiplying both sides by $u-u_{\alpha }$ and integrating by parts over Ω, we have:

$$\nu {∥\nabla \left(u-u_{\alpha }\right)∥}_{L^{2}\left(\Omega \right)}^{2}=\int_{\Gamma }\left(\nu \frac{\partial u}{\partial n}-pn\right)\left(u-u_{\alpha }\right)dx-\nu \alpha \int_{\Gamma }{\left(u-u_{\alpha }\right)}^{2}dx.$$

Since $\nu \alpha \int_{\Gamma }{\left(u-u_{\alpha }\right)}^{2}dx>0$, from the trace theorem [30], we have:

(8) $$\nu {∥\nabla \left(u-u_{\alpha }\right)∥}_{L^{2}\left(\Omega \right)}^{2}\leq C\left({∥u∥}_{H^{2}\left(\Omega \right)}+{∥p∥}_{H^{1}\left(\Omega \right)}\right){∥u-u_{\alpha }∥}_{L^{2}\left(\Gamma \right)}.$$

Combining (2) and Lemma 1 completes the proof. □

It can be found that when the penalty factor α approaches infinity, the solution $u_{\alpha }$ tends to u with $H^{1}$ semi-norm in the problem domain by Lemma 2. Lemmas 1 and 2 fully demonstrate the validity of the penalty method in a continuous sense.

## 4. Modified Weak Form for Penalized Stokes Problem

We define:

(9) $$X_{\alpha }\left(\Omega \right)=\left\{v\in {\left(H^{1}\left(\Omega \right)\right)}^{2}:\int_{\Gamma }\left(\nu \frac{\partial v}{\partial n}-pn+\alpha \nu v\right)vdx=0,{\left|v\right|}_{\alpha }<\infty \right\},$$

where

$${\left|v\right|}_{\alpha }^{2}=\int_{\Omega }{\left(\nabla v\right)}^{2}dx+\alpha \int_{\Gamma }v^{2}dx.$$

Clearly, applying Friedrich’s inequality yields:

(10) $$C{∥v∥}_{H^{1}\left(\Omega \right)}\leq {\left|v\right|}_{\alpha }.$$

**Lemma** **3.** *Let 0<h<diam(Ω) and a further assumption on $X_{\alpha }$ be:*

(11) $$h\int_{\Gamma }{\left(\nu \frac{\partial v}{\partial n}-p\cdot n\right)}^{2}dx\leq C{∥\nabla v∥}_{L^{2}\left(\Omega \right)}^{2},\;\forall v\in X_{\alpha }.$$

*When the penalty factor $\alpha >h^{-1}$, then:*

(12) $$C{∥v∥}_{H^{1}\left(\Omega \right)}\leq {\left|v\right|}_{\alpha }\leq C_{\nu }{∥v∥}_{H^{1}\left(\Omega \right)}.$$

**Proof.**  For any $v\in X_{\alpha }$, using assumption (11), we have:

(13) $$\nu^{2}\alpha^{2}\int_{\Gamma }v^{2}dx=\int_{\Gamma }{\left(\nu \frac{\partial v}{\partial n}-p\cdot n\right)}^{2}dx\leq \frac{C}{h}{∥\nabla v∥}_{L^{2}\left(\Omega \right)}^{2}.$$

If $\alpha >h^{-1}$, one gets:

(14) $$\alpha \int_{\Gamma }v^{2}dx\leq \frac{C}{\nu^{2}}{∥\nabla v∥}_{L^{2}\left(\Omega \right)}^{2},$$

then,

$${\left|v\right|}_{\alpha }^{2}=\int_{\Omega }{\left(\nabla v\right)}^{2}dx+\alpha \int_{\Gamma }v^{2}dx\leq \left(1+\frac{C}{\nu^{2}}\right){∥v∥}_{H^{1}\left(\Omega \right)}^{2}={\left(C_{\nu }{∥v∥}_{H^{1}\left(\Omega \right)}\right)}^{2},$$

which together with (10) implies (12). □

A discrete assumption similar to (11) is used in the Nitsche method to ensure the continuity and coercivity of the bilinear form for the elliptic equation [1]. In this paper, the assumption (11) is proposed to certify the continuity of $a_{\alpha }\left(\cdot ,\cdot \right)$ and inf-sup condition, thus deriving that $A_{\alpha }\left(\left(\cdot ,\cdot \right);\left(\cdot ,\cdot \right)\right)$ is continuous and coercive.

The modified weak form of (5) is to find $\left(u_{\alpha },p\right)\in X_{\alpha }\times M$ such that:

(15) $$A_{\alpha }\left(\left(u_{\alpha },p\right);\left(v,q\right)\right)=F\left(v\right),\;\;\forall \left(v,q\right)\in X_{\alpha }\times M,$$

where

(16) $$A_{\alpha }\left(\left(u_{\alpha },p\right);\left(v,q\right)\right)=a_{\alpha }\left(u_{\alpha },v\right)+b\left(v,p\right)+b\left(u_{\alpha },q\right),$$

(17) $$a_{\alpha }\left(u_{\alpha },v\right)=a\left(u_{\alpha },v\right)+\alpha \int_{\Gamma }u_{\alpha }vdx.$$

Clearly, using Lemma 3, the continuity of $a_{\alpha }\left(\cdot ,\cdot \right)$ follows:

$$\begin{array}{cc} \left|a_{\alpha }\left(v_{1},v_{2}\right)\right|\leq & \nu {∥v_{1}∥}_{H^{1}\left(\Omega \right)}{∥v_{2}∥}_{H^{1}\left(\Omega \right)}+\alpha {∥v_{1}∥}_{L^{2}\left(\Gamma \right)}{∥v_{1}∥}_{L^{2}\left(\Gamma \right)}\\ \leq & \max\left\{1,\nu \right\}{\left|v_{1}\right|}_{\alpha }{\left|v_{2}\right|}_{\alpha }\leq C{∥v_{1}∥}_{H^{1}\left(\Omega \right)}{∥v_{2}∥}_{H^{1}\left(\Omega \right)}. \end{array}$$

The coercivity of $a_{\alpha }\left(\cdot ,\cdot \right)$ follows:

$$a_{\alpha }\left(v,v\right)=\nu {∥v∥}_{H^{1}\left(\Omega \right)}^{2}+\alpha {∥v∥}_{L^{2}\left(\Gamma \right)}^{2}\geq \min\left\{1,\nu \right\}{\left|v\right|}_{\alpha }^{2}\geq C{∥v∥}_{H^{1}\left(\Omega \right)}^{2}.$$

Similarly, $b\left(\cdot ,\cdot \right)$ is continuous on $X_{\alpha }\times M$. Moreover, $b\left(\cdot ,\cdot \right)$ also satisfies the inf-sup condition,

$$\underset{v\in X_{\alpha },v\neq 0}{\sup}\frac{\left|b\left(v,q\right)\right|}{\;{\left|v\right|}_{\alpha }}\geq \underset{v\in X_{\alpha },v\neq 0}{\sup}\frac{\left|b\left(v,q\right)\right|}{\;C_{\nu }{∥v∥}_{H^{1}\left(\Omega \right)}}\geq \beta_{0}{∥q∥}_{L^{2}\left(\Omega \right)},\;\forall q\in M,$$

where $\beta_{0}=\beta /C_{\nu }$. Similar to $A\left(\left(\cdot ,\cdot \right);\left(\cdot ,\cdot \right)\right)$, $A_{\alpha }\left(\left(\cdot ,\cdot \right);\left(\cdot ,\cdot \right)\right)$ satisfies the continuity and coercivity conditions. Therefore, based on the Lax-Milgram theorem, (5) has a unique weak solution $\left(u_{\alpha },p\right)\in X_{\alpha }\times M$.

## 5. EFG for Penalized Stokes Problem

To approximate the solution of the modified weak form (15), the approximate space of the velocity is defined as:

(18) $$X_{h}=\left\{u_{h}\left(x\right)=\sum_{i=1}^{N_{1}}u\left(x_{i}\right)\Phi_{i}\left(x\right),u\left(x_{i}\right)\in R^{2}:\int_{\Gamma }\left(\nu \frac{\partial u_{h}}{\partial n}-p_{h}n+\alpha \nu u_{h}\right)u_{h}dx=0\right\},$$

and the approximate space of the pressure is:

(19) $$M_{h}=\left\{p_{h}\left(x\right)=\sum_{j=1}^{N_{2}}p\left(x_{j}\right)\Psi_{j}\left(x\right),p\left(x_{j}\right)\in R:\int_{\Omega }p_{h}\left(x\right)dx=0\right\},$$

in which ${\left\{x_{i}\right\}}_{i=1}^{N_{1}}$ is a set of $N_{1}$ velocity nodes in $\bar{\Omega }=\Omega \cup \Gamma$, ${\left\{x_{i}\right\}}_{i=1}^{N_{2}}$ is a set of $N_{2}$ pressure nodes. $\Phi_{i}\left(x\right)$ and $\Psi_{j}\left(x\right)$ represent the MLS shape functions based on velocity nodes and pressure nodes, respectively.

Now, we briefly state the MLS shape function and its approximation error by taking the velocity nodes as an example, which is similar to the pressure nodes. The MLS shape functions $\Phi_{i}\left(x\right)$ are:

(20) $$\Phi_{i}\left(x\right)=\left\{\begin{array}{cc} \sum_{j=1}^{m}p_{j}\left(x\right){\left[A^{-1}\left(x\right)B\left(x\right)\right]}_{jI}, & i=\lambda_{I}\in \wedge \left(x\right),\\ 0, & i\notin \wedge \left(x\right), \end{array}\right.\;i=1,2,\cdots ,N_{1},$$

in which $p_{j}\left(x\right)$ denotes the shifted and scaled monomial basis function [22,24,25,28], $\wedge \left(x\right)=\left\{\lambda_{1},\lambda_{2},\cdots ,\lambda_{n_{x}}\right\}$ means the global sequence numbers of nodes whose support domains cover the point x. The support domain of x is $ℜ\left(x\right)$ with radius $r\left(x\right)$, $ℜ\left(x\right)=\left\{y\in R^{2}:\left|y-x\right|\leq r\left(x\right)\right\}$. $A\left(x\right)=P^{T}W\left(x\right)P$, $B\left(x\right)=P^{T}W\left(x\right)$, $P={\left(p\left(x_{\lambda_{1}}\right),\cdots ,p\left(x_{\lambda_{n_{x}}}\right)\right)}^{T}$, $p\left(x_{i}\right)={\left(p_{1}\left(x_{i}\right),\cdots ,p_{m}\left(x_{i}\right)\right)}^{T}$ and $W\left(x\right)=\mathrm{diag}\left(w_{\lambda_{1}}\left(x\right),\cdots ,w_{\lambda_{n_{x}}}\left(x\right)\right)$ with weight function $w_{i}\left(x\right)$.

Assume that the configuration of velocity nodes ${\left\{x_{i}\right\}}_{i=1}^{N_{1}}$ satisfies the following conditions:

(B1) Define the characteristic distance *h* as
  $$h\leq h_{i}\leq Ch,\;h_{i}=\min_{1\leq j\leq N_{1},j\neq i}\left|x_{i}-x_{j}\right|.$$
(B2) To ensure the invertibility of $A\left(x\right)$,
  $$\mathrm{card}\left\{\wedge \left(x\right)\right\}\geq \dim\left\{p_{j}\left(x\right)\right\}=\frac{\left(\hat{m}+2\right)\left(\hat{m}+1\right)}{2},$$
  where $\hat{m}$ represents the largest degree of the used monomial basis functions.

Moreover, assume that derivatives of the weight function $w_{i}\left(x\right)$ up to order γ are bounded and continuous such that $w_{i}\left(x\right)\in C_{0}^{\gamma }\left(ℜ\left(x_{i}\right)\right)$. Then, MLS shape functions $\Phi_{i}\left(x\right)$ are bounded and γ-times continuously differentiable, i.e., $\Phi_{i}\left(x\right)\in C_{0}^{\gamma }\left(ℜ\left(x_{i}\right)\right)$.

**Lemma** **4** ([24])**.** *Assume that $w\in H^{\hat{m}+1}\left(\Omega \right)$, conditions (B1) and (B2) are satisfied, Mw denotes the MLS approximation of w. Then*

$${∥w-Mw∥}_{H^{k}\left(\Omega \right)}\leq Ch^{\hat{m}+1-k}{∥w∥}_{H^{\hat{m}+1}\left(\Omega \right)},\;0\leq k\leq \min\left\{\hat{m}+1,\gamma \right\}.$$

The following lemma is regarded as a sufficient condition for $\left(X_{h},M_{h}\right)$ to satisfy the discrete inf-sup condition in the meshless method.

**Lemma** **5** ([9,10])**.** *Assume that $\left(X_{h},M_{h}\right)$ satisfies the following condition, for any $i\in \theta_{j}=\left\{l:x_{l}\in \mathrm{supp}\left(\Phi_{j}\right)\right\}$, $j=1,2,\cdots ,N_{2},$*

$$\left|\int_{\Omega }\Psi_{j}\frac{\partial \Phi_{i}}{\partial x_{\eta }}dx\right|-\sum_{k\in \pi_{j}}\left|\int_{\Omega }\Psi_{k}\frac{\partial \Phi_{i}}{\partial x_{\eta }}dx\right|\geq Ch,$$

*where η=1,2, the index set $\pi_{j}=\left\{l\neq j:\mathrm{supp}\left(\Psi_{l}\right)\cap \mathrm{supp}\left(\Psi_{j}\right)\neq \emptyset \right\}$. Then $\left(X_{h},M_{h}\right)$ satisfies the discrete inf-sup condition*

(21) $$\underset{v\in X_{h},v\neq 0}{\sup}\frac{\left|b\left(v,q\right)\right|}{\;{∥v∥}_{H^{1}\left(\Omega \right)}}\geq \beta_{1}{∥q∥}_{L^{2}\left(\Omega \right)},\;\forall q\in M_{h},$$

*where $\beta_{1}$ is independent of h.*

The EFG method for (15) is to find $\left(u_{h},p_{h}\right)\in \left(X_{h},M_{h}\right)$ such that:

(22) $$\begin{array}{c} \left\{\begin{array}{c} a_{\alpha }\left(u_{h},v\right)+b\left(v,p_{h}\right)=F\left(v\right),\;\forall v\in X_{h},\\ b\left(u_{h},q\right)=0,\;\forall q\in M_{h}. \end{array}\right. \end{array}$$

The EFG solutions $u_{h}$ and $p_{h}$ have an estimate similar to Lemma 1.

**Lemma** **6.** *Assume that (22) satisfies the following regularity:*

(23) $${∥u_{h}∥}_{H^{2}\left(\Omega \right)}+{∥p_{h}∥}_{H^{1}\left(\Omega \right)}\leq C{∥f∥}_{L^{2}\left(\Omega \right)}.$$

*Then,*

(24) $${∥u_{h}∥}_{L^{2}\left(\Gamma \right)}\leq \frac{C}{\alpha }.$$

**Proof.**  Combining the definition of (18) and the trace theorem [30],

$$\alpha \nu {∥u_{h}∥}_{L^{2}\left(\Gamma \right)}^{2}\leq {∥p_{h}n-\nu \frac{\partial u_{h}}{\partial n}∥}_{L^{2}\left(\Gamma \right)}{∥u_{h}∥}_{L^{2}\left(\Gamma \right)}\leq C\left({∥p_{h}∥}_{H^{1}\left(\Omega \right)}+{∥u_{h}∥}_{H^{2}\left(\Omega \right)}\right){∥u_{h}∥}_{L^{2}\left(\Gamma \right)},$$

which together with (23) implies (24). □

## 6. Error Analysis

First of all, an error bound for velocity in the $H^{1}\left(\Omega \right)$ norm and an error bound for pressure in the $L^{2}\left(\Omega \right)$ norm are given separately.

**Theorem** **1.** *Let $\left(u,p\right)\in {\left(H^{\hat{m}+1}\left(\Omega \right)\right)}^{2}\times \left(H^{\hat{m}}\left(\Omega \right)\cap M\right)$ and $\left(u_{h},p_{h}\right)$ be the solutions of (1) and (22), respectively. Assume that $u_{\alpha }\in {\left(H^{\hat{m}+1}\left(\Omega \right)\right)}^{2}$ and *Γ* is sufficiently smooth, then:*

(25) $${∥u-u_{h}∥}_{H^{1}\left(\Omega \right)}\leq C\left(h^{\hat{m}}{∥u_{\alpha }∥}_{H^{\hat{m}+1}\left(\Omega \right)}+\sqrt{\alpha }h^{\hat{m}+1/2}{∥u_{\alpha }∥}_{H^{\hat{m}+1}\left(\Omega \right)}+h^{\hat{m}}{∥p∥}_{H^{\hat{m}}\left(\Omega \right)}+\alpha^{-1}\right),$$

(26) $${∥p-p_{h}∥}_{L^{2}\left(\Omega \right)}\leq C\left(h^{\hat{m}}{∥u_{\alpha }∥}_{H^{\hat{m}+1/2}\left(\Omega \right)}+\sqrt{\alpha }h^{\hat{m}+1/2}{∥u_{\alpha }∥}_{H^{\hat{m}+1}\left(\Omega \right)}+h^{\hat{m}}{∥p∥}_{H^{\hat{m}}\left(\Omega \right)}\right).$$

**Proof.**  Subtracting (15) from (22) yields:

(27) $$\begin{array}{c} \left\{\begin{array}{c} a_{\alpha }\left(u_{\alpha }-u_{h},v\right)+b\left(v,p-p_{h}\right)=0,\;\forall v\in X_{h},\\ b\left(u_{\alpha }-u_{h},q\right)=0,\;\forall q\in M_{h}. \end{array}\right. \end{array}$$

Choosing $v=u_{h}-Mu_{\alpha }$ in the first equation of the above formula yields:

(28) $$\begin{array}{cc} a_{\alpha }\left(u_{\alpha }-u_{h},u_{\alpha }-u_{h}\right)= & a_{\alpha }\left(u_{\alpha }-u_{h},u_{\alpha }-Mu_{\alpha }\right)+b\left(u_{h}-Mu_{\alpha },p-p_{h}\right)\\ = & a_{\alpha }\left(u_{\alpha }-u_{h},u_{\alpha }-Mu_{\alpha }\right)+b\left(u_{h},p-p_{h}\right)-b\left(Mu_{\alpha },p-p_{h}\right)\\ = & I_{1}+I_{2}-I_{3}. \end{array}$$

Applying the continuity of $a_{\alpha }\left(\cdot ,\cdot \right)$ gets:

(29) $$\begin{array}{cc} I_{1}\leq & {\left|u_{\alpha }-u_{h}\right|}_{\alpha }{\left|u_{\alpha }-Mu_{\alpha }\right|}_{\alpha }\\ \leq & \frac{{\left|u_{\alpha }-u_{h}\right|}_{\alpha }^{2}}{4}+{\left|u_{\alpha }-Mu_{\alpha }\right|}_{\alpha }^{2}. \end{array}$$

Since $Mp-p_{h}\in M_{h}$ and $\nabla \cdot u_{\alpha }=0$, we have:

(30) $$\begin{array}{cc} I_{2}= & b\left(u_{h},p-Mp+Mp-p_{h}\right)\\ = & b\left(u_{h},p-Mp\right)+b\left(u_{h},Mp-p_{h}\right)\\ = & b\left(u_{h}-u_{\alpha },p-Mp\right)+b\left(u_{h}-u_{\alpha },Mp-p_{h}\right)\\ = & b\left(u_{h}-u_{\alpha },p-Mp\right)\\ \leq & {∥\nabla \left(u_{h}-u_{\alpha }\right)∥}_{L^{2}\left(\Omega \right)}{∥p-Mp∥}_{L^{2}\left(\Omega \right)}\\ \leq & \frac{{\left|u_{h}-u_{\alpha }\right|}_{\alpha }^{2}}{4}+{∥p-Mp∥}_{L^{2}\left(\Omega \right)}^{2}. \end{array}$$

Again using $\nabla \cdot u_{\alpha }=0$ obtains:

(31) $$\begin{array}{cc} I_{3}= & b\left(Mu_{\alpha },p-p_{h}\right)\\ = & b\left(Mu_{\alpha }-u_{\alpha },p-p_{h}\right)\\ \leq & {∥\nabla \left(Mu_{\alpha }-u_{\alpha }\right)∥}_{L^{2}\left(\Omega \right)}{∥p-p_{h}∥}_{L^{2}\left(\Omega \right)}\\ \leq & {∥\nabla \left(Mu_{\alpha }-u_{\alpha }\right)∥}_{L^{2}\left(\Omega \right)}\left({∥p-Mp∥}_{L^{2}\left(\Omega \right)}+{∥Mp-p_{h}∥}_{L^{2}\left(\Omega \right)}\right). \end{array}$$

Combining the discrete inf-sup condition (21) and (27), there exists a $\zeta \in X_{h}$ such that:

(32) $$\begin{array}{cc} \beta_{1}{∥p_{h}-Mp∥}_{L^{2}\left(\Omega \right)}\leq & \frac{b\left(\zeta ,p_{h}-Mp\right)}{{∥\zeta ∥}_{H^{1}\left(\Omega \right)}}=\frac{a_{\alpha }\left(u_{\alpha }-u_{h},\zeta \right)+b\left(\zeta ,p-Mp\right)}{{∥\zeta ∥}_{H^{1}\left(\Omega \right)}}\\ \leq & C{\left|u_{\alpha }-u_{h}\right|}_{\alpha }+{∥p-Mp∥}_{L^{2}\left(\Omega \right)}. \end{array}$$

Then,

(33) $$\begin{array}{cc} I_{3}\leq & {∥\nabla \left(Mu_{\alpha }-u_{\alpha }\right)∥}_{L^{2}\left(\Omega \right)}\left(\left(1+\frac{1}{\beta_{1}}\right){∥p-Mp∥}_{L^{2}\left(\Omega \right)}+\frac{C}{\beta_{1}}{\left|u_{\alpha }-u_{h}\right|}_{\alpha }\right)\\ \leq & \left(1+\frac{1}{\beta_{1}}\right){∥\nabla \left(Mu_{\alpha }-u_{\alpha }\right)∥}_{L^{2}\left(\Omega \right)}{∥p-Mp∥}_{L^{2}\left(\Omega \right)}+I_{3}^{0}, \end{array}$$

where

(34) $$\begin{array}{cc} I_{3}^{0}= & \frac{C}{\beta_{1}}{∥\nabla \left(Mu_{\alpha }-u_{\alpha }\right)∥}_{L^{2}\left(\Omega \right)}{\left|u_{\alpha }-u_{h}\right|}_{\alpha }\\ \leq & \frac{C}{\beta_{1}}\left(\delta {\left|u_{\alpha }-u_{h}\right|}_{\alpha }^{2}+\frac{{∥\nabla \left(Mu_{\alpha }-u_{\alpha }\right)∥}_{L^{2}\left(\Omega \right)}^{2}}{4\delta }\right). \end{array}$$

Inserting (29)–(34) into (28), considering δ<β1β1ν2C2C and Lemma 3 yields:

(35) $$\begin{array}{cc} {∥u_{\alpha }-u_{h}∥}_{H^{1}\left(\Omega \right)}\leq & C\left({\left|u_{\alpha }-Mu_{\alpha }\right|}_{\alpha }+{∥p-Mp∥}_{L^{2}\left(\Omega \right)}\right)\\ \leq & C\left(h^{\hat{m}}{∥u_{\alpha }∥}_{H^{\hat{m}+1}\left(\Omega \right)}+\sqrt{\alpha }{∥u_{\alpha }-Mu_{\alpha }∥}_{L^{2}\left(\Gamma \right)}+h^{\hat{m}}{∥p∥}_{H^{\hat{m}}\left(\Omega \right)}\right). \end{array}$$

From Lemma 3 and the trace inequality, we have:

(36) $${∥u_{\alpha }-Mu_{\alpha }∥}_{L^{2}\left(\Gamma \right)}^{2}\leq C{∥u_{\alpha }-Mu_{\alpha }∥}_{L^{2}\left(\Omega \right)}{∥u_{\alpha }-Mu_{\alpha }∥}_{H^{1}\left(\Omega \right)}\leq Ch^{2\hat{m}+1}{∥u_{\alpha }∥}_{H^{\hat{m}+1}\left(\Omega \right)}^{2}.$$

Therefore,

(37) $${∥u_{\alpha }-u_{h}∥}_{H^{1}\left(\Omega \right)}\leq C\left(h^{\hat{m}}{∥u_{\alpha }∥}_{H^{\hat{m}+1}\left(\Omega \right)}+\sqrt{\alpha }h^{\hat{m}+1/2}{∥u_{\alpha }∥}_{H^{\hat{m}+1}\left(\Omega \right)}+h^{\hat{m}}{∥p∥}_{H^{\hat{m}}\left(\Omega \right)}\right).$$

Using

(38) $${∥p-p_{h}∥}_{L^{2}\left(\Omega \right)}\leq {∥p-Mp∥}_{L^{2}\left(\Omega \right)}+{∥Mp-p_{h}∥}_{L^{2}\left(\Omega \right)},$$

together with (32) and (37) imply (26).

Let $w=w\left(x\right)\in {\left(H^{1}\left(\Omega \right)\right)}^{2}$ be the weak solution of:

$$\left\{\begin{array}{cc} & -\nu \Delta w=0,\;\mathrm{in}\;\Omega ,\\ & w=\nu \frac{\partial u}{\partial n}-pn,\;\mathrm{on}\;\Gamma , \end{array}\right.$$

and let $u=u_{\alpha }+\alpha^{-1}w+z$, then:

(39) $${∥u-u_{h}∥}_{H^{1}\left(\Omega \right)}\leq {∥u_{\alpha }-u_{h}∥}_{H^{1}\left(\Omega \right)}+\alpha^{-1}{∥w∥}_{H^{1}\left(\Omega \right)}+{∥z∥}_{H^{1}\left(\Omega \right)}.$$

By the definition of $a_{\alpha }\left(\cdot ,\cdot \right)$, the function z satisfies:

(40) $$a_{\alpha }\left(z,v\right)=a_{\alpha }\left(u,v\right)-a_{\alpha }\left(u_{\alpha },v\right)-\alpha^{-1}a_{\alpha }\left(w,v\right).$$

Combining Green’s formula and the fact ${u|}_{\Gamma }=0$ gives:

(41) $$\begin{array}{cc} a_{\alpha }\left(u,v\right)= & \nu \int_{\Omega }\nabla u\nabla udx+\alpha \int_{\Gamma }uvdx\\ = & -\nu \int_{\Omega }\Delta uvdx+\nu \int_{\Gamma }\frac{\partial u}{\partial n}vdx\\ = & \int_{\Omega }\left(f-\nabla p\right)vdx+\nu \int_{\Gamma }\frac{\partial u}{\partial n}vdx. \end{array}$$

(42) $$\begin{array}{cc} a_{\alpha }\left(u_{\alpha },v\right)= & \nu \int_{\Omega }\nabla u_{\alpha }\nabla udx+\alpha \int_{\Gamma }u_{\alpha }vdx\\ = & -\nu \int_{\Omega }\Delta u_{\alpha }vdx+\nu \int_{\Gamma }\frac{\partial u}{\partial n}vdx+\alpha \int_{\Gamma }u_{\alpha }vdx\\ = & \int_{\Omega }\left(f-\nabla p\right)vdx+\int_{\Gamma }pnvdx. \end{array}$$

(43) $$\begin{array}{cc} a_{\alpha }\left(w,v\right)= & \nu \int_{\Omega }\nabla w\nabla udx+\alpha \int_{\Gamma }wvdx\\ = & \nu \int_{\Omega }\nabla w\nabla vdx+\alpha \int_{\Gamma }\left(\nu \frac{\partial u}{\partial n}-pn\right)vdx. \end{array}$$

Inserting (41)–(43) into (40) and choosing v=z yield

$$a_{\alpha }\left(z,z\right)=-\alpha^{-1}\nu \int_{\Omega }\nabla w\nabla zdx.$$

Hence

$${∥z∥}_{H^{1}\left(\Omega \right)}^{2}\leq C{\left|z\right|}_{\alpha }^{2}\leq C\alpha^{-1}{∥w∥}_{H^{1}\left(\Omega \right)}{∥z∥}_{H^{1}\left(\Omega \right)},$$

which together with (37) and (39) implies (25). □

According to Lemmas 1 and 2, theoretically, the penalty method requires that the penalty factor α tends to infinity to impose the Dirichlet boundary condition. Nevertheless, in numerical calculations, the coefficient matrix of the resulting system will become ill-conditioned when the penalty factor increases uncontrollably. By deploying $\alpha =Ch^{\left(-2\hat{m}-1\right)/3}$ in (25) and (26), the optimal convergence rates are derived as:

(44) $${∥u-u_{h}∥}_{H^{1}\left(\Omega \right)}\leq Ch^{\frac{2\hat{m}+1}{3}},\;{∥p-p_{h}∥}_{L^{2}\left(\Omega \right)}\leq Ch^{\frac{2\hat{m}+1}{3}}.$$

When the linear basis function is chosen in the MLS approximation, i.e., $\hat{m}=1$ and the penalty factor $\alpha =Ch^{-1}$, the corresponding convergence rates are optimal as:

(45) $${∥u-u_{h}∥}_{H^{1}\left(\Omega \right)}\leq Ch,\;{∥p-p_{h}∥}_{L^{2}\left(\Omega \right)}\leq Ch.$$

Clearly, in this case, the EFG solution $u_{h}$ converges to the exact solution u with optimal convergence rate *h* in $H^{1}\left(\Omega \right)$, but the pressure numerical solution $p_{h}$ only maintains first order convergence in $L^{2}\left(\Omega \right)$.

To obtain the numerical error of velocity u in $L^{2}$ norm, the following definition and lemma are required.

**Definition** **1** ([25,31])**.** *Let $0<h_{0}\leq 1$ and $0\leq k_{1}\leq k_{0}$. A system of functions $q\in H^{k_{1}}\left(\Omega \right)$ called $\left(k_{0},k_{1}\right)$-regular functions and presented by $\gamma_{h_{0}}^{k_{0},k_{1}}\left(\Omega \right)$ if and only if, for any $w\in H^{l}\left(\Omega \right)$, there is a function $\xi \in \gamma_{h_{0}}^{k_{0},k_{1}}\left(\Omega \right)$ such that:*

$${∥w-\xi ∥}_{H^{l_{0}}\left(\Omega \right)}\leq Ch_{0}^{l_{1}}{∥w∥}_{H^{l}\left(\Omega \right)},\;0\leq l_{0}\leq \min\left\{l,k_{1}\right\},$$

*in which $l_{1}=\min\left\{l-l_{0},k_{0}-l_{0}\right\}$. If $w\in H^{l}\left(\Omega \right)$ has a compact support $\Omega_{s}$, then ξ has a compact support $\Omega_{s}^{\rho }$ such that:*

$$\Omega_{s}^{\rho }\subset \left\{x\in \Omega :\mathrm{dist}\left(x,\Omega_{s}\right)\leq Ch_{0}\right\},$$

*where $\mathrm{dist}\left(x,\Omega_{s}\right)$ denotes the distance from x to $\Omega_{s}$.*

The following approximate error follows from the above definition.

**Lemma** **7** ([25,31])**.** *Let $w\in H^{l}\left(\Omega \right)$ and w=0 on *Γ*. If *Γ* is sufficiently smooth, $k_{0}\geq l\geq 2$ and $k_{1}\geq 1$, then there exists a function $\xi \in \gamma_{h_{0}}^{k_{0},k_{1}}\left(\Omega \right)$ such that:*

w−ξHμΩ+Ch0−ε−ε22ξL2Γ≤Ch0κwHlΩ,

*in which $0\leq \mu \leq k_{1}$, ε>0, κ=k0−t0l−μk0−t0l−μk0−μk0−μ and t0=maxμ,1+ε1+ε22.*

Clearly, the MLS shape functions satisfy the requirements of ξ in Definition 1. Therefore, $X_{h}\times M_{h}\subset {\left(\gamma_{h}^{k_{0},k_{1}}\left(\Omega \right)\right)}^{2}\times \gamma_{h}^{k_{0},k_{1}}\left(\Omega \right)$ with $k_{0}\geq \hat{m}+1$ and $k_{1}\geq 1$. Now, with the aid of the duality argument, an error bound of u in the $L^{2}$ norm can be derived.

**Theorem** **2.** *Let $u\in {\left(H^{\hat{m}+1}\left(\Omega \right)\right)}^{2}$ and $u_{h}$ be the solutions of (1) and (22), respectively. Assume that $u_{\alpha }\in {\left(H^{\hat{m}+1}\left(\Omega \right)\right)}^{2}$, *Γ* is sufficiently smooth and $\alpha =Ch^{-\sigma }$, then:*

(46) $$\begin{array}{cc} {∥u-u_{h}∥}_{L^{2}\left(\Omega \right)}\leq & C\left(h^{\kappa_{1}}+h^{\kappa_{2}}\right)\left(\left(h^{\hat{m}}+\sqrt{\alpha }h^{\hat{m}+1/2}\right){∥u_{\alpha }∥}_{H^{\hat{m}+1}\left(\Omega \right)}+h^{\hat{m}}{∥p∥}_{H^{\hat{m}}\left(\Omega \right)}\right)\\ + & C\left(\sqrt{\alpha^{-1}}h^{\kappa_{1}}+\alpha^{-1}\right)+C\left(h^{\kappa_{1}}+h^{\kappa_{2}}\right)\alpha^{-1}, \end{array}$$

*where $\kappa_{1}=\frac{k_{0}-t_{0}}{k_{0}-1}$ with $t_{0}=\max\left\{1,\frac{1+\sigma }{2}\right\}$, and $\kappa_{2}=\frac{2\left(k_{0}-t_{1}\right)}{k_{0}}$ with $t_{1}=\frac{1+\sigma }{2}$. Considering the case of $k_{0}$ is large enough, we obtain:*

(47) $${∥u-u_{h}∥}_{L^{2}\left(\Omega \right)}\leq C\left(h\left(\left(h^{\hat{m}}+\sqrt{\alpha }h^{\hat{m}+1/2}\right){∥u_{\alpha }∥}_{H^{\hat{m}+1}\left(\Omega \right)}+h^{\hat{m}}{∥p∥}_{H^{\hat{m}}\left(\Omega \right)}+\sqrt{\alpha^{-1}}\right)+\alpha^{-1}\right).$$

**Proof.**  Define the error $e_{h}=u-u_{h}$. For any $\left(j,s\right)\in X\times M$, we have:

(48) $$A\left(\left(j,s\right);\left(v,q\right)\right)=\left(e_{h},v\right),\;\left(v,q\right)\in X\times M.$$

Moreover, assume that the solution of (48) satisfies:

(49) $${∥j∥}_{H^{2}\left(\Omega \right)}+{∥s∥}_{H^{1}\left(\Omega \right)}\leq C{∥e_{h}∥}_{L^{2}\left(\Omega \right)}.$$

Define the error $e_{p}=p-p_{h}$,

(50) $$A\left(\left(e_{h},e_{p}\right);\left(v,q\right)\right)-\alpha \int_{\Gamma }\left(\left(\nu \frac{\partial u}{\partial n}-pn\right)\frac{1}{\alpha }-e_{h}\right)vdx=0,\;\left(v,q\right)\in {\left(H^{1}\left(\Omega \right)\right)}^{2}\times M.$$

According to Lemma 7, choosing $\alpha =Ch^{-\sigma }$, there exists $g_{h}\in {\left(\gamma_{h}^{k_{0},k_{1}}\right)}^{2}$ and $m_{h}\in \gamma_{h}^{k_{0},k_{1}}$ such that:

(51) $${∥j-g_{h}∥}_{H^{1}\left(\Omega \right)}+\sqrt{\alpha }{∥g_{h}∥}_{L^{2}\left(\Gamma \right)}\leq Ch^{\kappa_{1}}{∥j∥}_{H^{2}\left(\Omega \right)}\leq Ch^{\kappa_{1}}{∥e_{h}∥}_{L^{2}\left(\Omega \right)},$$

(52) $${∥s-m_{h}∥}_{L^{2}\left(\Omega \right)}+\sqrt{\alpha }{∥m_{h}∥}_{L^{2}\left(\Gamma \right)}\leq Ch^{\kappa_{2}}{∥s∥}_{H^{1}\left(\Omega \right)}\leq Ch^{\kappa_{2}}{∥e_{h}∥}_{L^{2}\left(\Omega \right)},$$

where $\kappa_{1}=\frac{k_{0}-t_{0}}{k_{0}-1}$ with $t_{0}=\max\left\{1,\frac{1+\sigma }{2}\right\}$, and $\kappa_{2}=\frac{2\left(k_{0}-t_{1}\right)}{k_{0}}$ with $t_{1}=\frac{1+\sigma }{2}$. Since $\left(e_{h},e_{p}\right)\left|{}_{\Gamma }\right.\neq \left(0,0\right)$, taking $\left(v,q\right)=\left(e_{h},e_{p}\right)$ in (48) yields

(53) $$A\left(\left(j,s\right);\left(e_{h},e_{p}\right)\right)=\left(e_{h},e_{h}\right)+\int_{\Gamma }\left(\nu \frac{\partial j}{\partial n}-sn\right)e_{h}dx.$$

Again, choosing $\left(v,q\right)=\left(g_{h},m_{h}\right)$ in (50) provides:

(54) $$A\left(\left(e_{h},e_{p}\right);\left(g_{h},m_{h}\right)\right)=\int_{\Gamma }\left(\nu \frac{\partial u}{\partial n}-pn\right)g_{h}dx-\alpha \int_{\Gamma }e_{h}g_{h}dx.$$

In addition,

(55) $$A\left(\left(e_{h},e_{p}\right);\left(g_{h},m_{h}\right)\right)=A\left(\left(j,s\right);\left(e_{h},e_{p}\right)\right)+A\left(\left(g_{h}-j,m_{h}-s\right);\left(e_{h},e_{p}\right)\right).$$

Inserting (54) and (55) into (53) gets:

(56) $$\begin{array}{cc} {∥e_{h}∥}_{L^{2}\left(\Omega \right)}^{2}\leq & \left|A\left(\left(g_{h}-j,m_{h}-s\right);\left(e_{h},e_{p}\right)\right)\right|+\left|\int_{\Gamma }\left(\nu \frac{\partial j}{\partial n}-sn\right)e_{h}dx\right|\\ + & \alpha \left|\int_{\Gamma }e_{h}g_{h}dx\right|+\left|\int_{\Gamma }\left(\nu \frac{\partial u}{\partial n}-pn\right)g_{h}dx\right|\\ = & J_{0}+J_{1}+J_{2}+J_{3}. \end{array}$$

Furthermore, combining Lemma 6, (51) and (52) leads to:

(57) $$J_{0}\leq C\left(h^{\kappa_{1}}+h^{\kappa_{2}}\right)\left({∥e_{h}∥}_{H^{1}\left(\Omega \right)}+{∥e_{p}∥}_{L^{2}\left(\Omega \right)}\right){∥e_{h}∥}_{L^{2}\left(\Omega \right)},$$

(58) $$J_{1}\leq C{∥e_{h}∥}_{L^{2}\left(\Gamma \right)}\left({∥j∥}_{H^{2}\left(\Omega \right)}+{∥s∥}_{H^{1}\left(\Omega \right)}\right),$$

(59) $$J_{2}\leq \alpha {∥e_{h}∥}_{L^{2}\left(\Gamma \right)}{∥g_{h}∥}_{L^{2}\left(\Gamma \right)}\leq C\sqrt{\alpha^{-1}}h^{\kappa_{1}}{∥e_{h}∥}_{L^{2}\left(\Omega \right)}.$$

(60) $$J_{3}\leq C\sqrt{\alpha^{-1}}h^{\kappa_{1}}{∥e_{h}∥}_{L^{2}\left(\Omega \right)}\left({∥u∥}_{H^{2}\left(\Omega \right)}+{∥p∥}_{H^{1}\left(\Omega \right)}\right).$$

Inserting (57)–(60) into (56) yields:

(61) $${∥e_{h}∥}_{L^{2}\left(\Omega \right)}\leq C\left(\left(h^{\kappa_{1}}+h^{\kappa_{2}}\right)\left({∥e_{h}∥}_{H^{1}\left(\Omega \right)}+{∥e_{p}∥}_{L^{2}\left(\Omega \right)}\right)+\sqrt{\alpha^{-1}}h^{\kappa_{1}}+\alpha^{-1}\right),$$

which together with Theorem 1 implies (46). As in Refs. [25,31], (47) is obtained for $k_{0}$ as sufficiently large. □

Substituting $\alpha =Ch^{-\sigma }$ into (47), we have:

u−uhL2Ω≤Chm^+1uαHm^+1Ω+hm^+3/2−σσ22uαHm^+1Ω+hm^+1pHm^+h1+σ/2+hσ.

Therefore, as suggested by Theorem 1, for linear basis function, when σ=1, the convergence rate is:

(62) $${∥u-u_{h}∥}_{L^{2}\left(\Omega \right)}\leq Ch.$$

Furthermore, when $\sigma =\frac{5}{3}$, we can obtain a suboptimal convergence rate as:

(63) $${∥u-u_{h}∥}_{L^{2}\left(\Omega \right)}\leq Ch^{1+\frac{2}{3}}.$$

## 7. Numerical Examples

**Figure 1:**
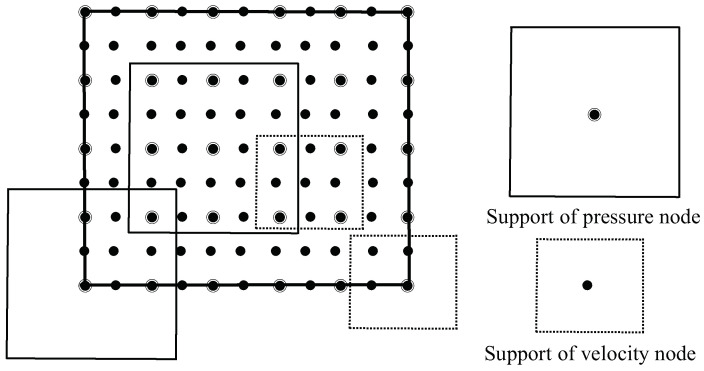
Configuration for velocity and pressure nodes.

This section presents four numerical examples to illustrate the theoretical error results proposed in the previous section. The problem domain is the unit square $\Omega =\left[0,1\right]\times \left[0,1\right]$. An efficient discrete node configuration for velocity and pressure has been proposed to satisfy the condition of Lemma 5 [9,10], see Figure 1.

### 7.1. Example 1

The first example is the Stokes problem (1) with the viscosity ν=1. The exact solution is:

$$u_{1}=-256x_{1}^{2}{\left(x_{1}-1\right)}^{2}x_{2}\left(x_{2}-1\right)\left(2x_{2}-1\right),$$

$$u_{2}=256x_{2}^{2}{\left(x_{2}-1\right)}^{2}x_{1}\left(x_{1}-1\right)\left(2x_{1}-1\right),$$

$$p=150\left(x_{1}-0.5\right)\left(x_{2}-0.5\right).$$

**Figure 2:**
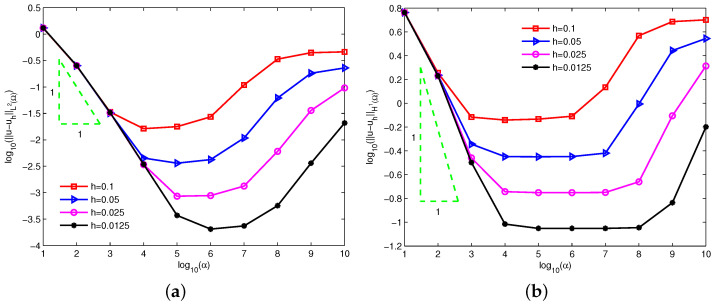
Influence of the increasing penalty factors α on errors (**a**) ${∥u-u_{h}∥}_{L^{2}\left(\Omega \right)}$ and (**b**) ${∥u-u_{h}∥}_{H^{1}\left(\Omega \right)}$ for example 1.

Figure 2 depicts the log-log plots of the errors ${∥u-u_{h}∥}_{L^{2}\left(\Omega \right)}$ and ${∥u-u_{h}∥}_{H^{1}\left(\Omega \right)}$ with respect to increasing penalty factors $\alpha =10,10^{2},10^{3},\cdots ,10^{9},10^{10}$ for linear basis function ($\hat{m}=1$). The radius of support domain of the velocity node is 1.5h and four types of equidistant nodes 11×11, 21×21, 41×41 and 81×81 are used. Clearly, a too-small or too-big penalty factor increases the numerical errors and leads to the invalidation of numerical calculations. Theorems 1 and 2 imply that the theoretical errors of velocity are dominated by $\alpha^{-1}$ for a small penalty factor. It can be observed that the numerical errors of velocity keep almost the same convergence order $\alpha^{-1}$ from the left sides of Figure 2a,b. Obviously, the numerical errors of velocity agree well with the theoretical error estimates.

**Figure 3:**
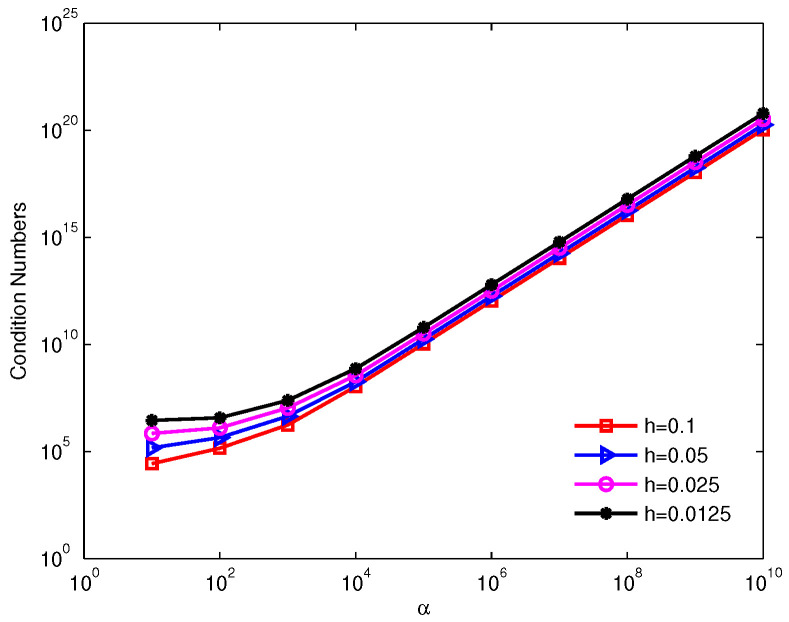
Condition numbers of the discrete coefficient matrix for the increasing penalty factors for example 1.

The condition numbers of the discrete coefficient matrix for the increasing penalty factors are shown in Figure 3. It is clear that the condition numbers increase with the increase of the penalty factor and the condition number is approximately $\alpha^{2}$. Therefore, a too-big penalty factor predestinates invalidate the penalty method, which in turn leads to the failure of the numerical methods using the penalty method.

**Figure 4:**
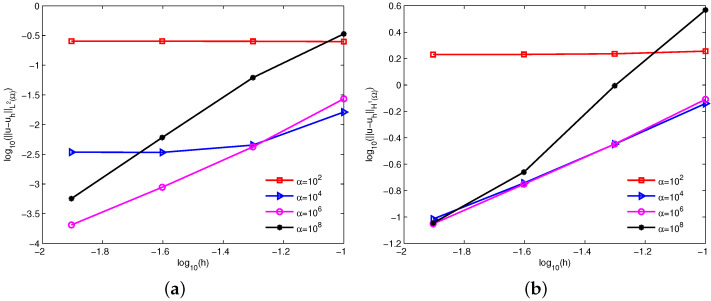
Influence of the constant penalty factors α on errors (**a**) ${∥u-u_{h}∥}_{L^{2}\left(\Omega \right)}$ and (**b**) ${∥u-u_{h}∥}_{H^{1}\left(\Omega \right)}$ for example 1.

Figure 4 reveals the log-log plots of the errors ${∥u-u_{h}∥}_{L^{2}\left(\Omega \right)}$ and ${∥u-u_{h}∥}_{H^{1}\left(\Omega \right)}$ with respect to the nodal spacing *h* for the constant penalty factors $\alpha =10^{2},10^{4},10^{6},10^{8}$. Clearly, as *h* is halved, the errors hardly change for a too-small penalty factor and decrease for some large penalty factors. These numerical errors are in line with the theoretical analysis.

**Figure 5:**
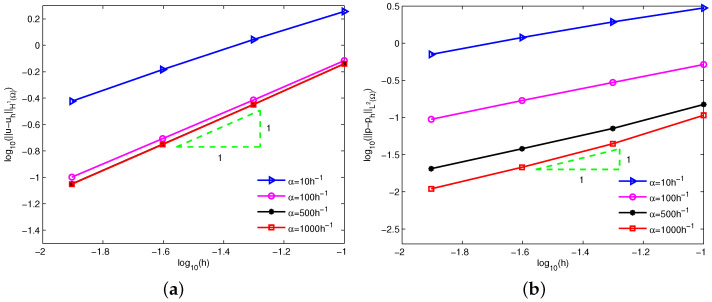
Errors of (**a**) ${∥u-u_{h}∥}_{H^{1}\left(\Omega \right)}$ and (**b**) ${∥p-p_{h}∥}_{L^{2}\left(\Omega \right)}$ for different constant $C_{s}$ for example 1.

From the point of view of the numerical results above, a suitably large constant penalty factor can obtain stable numerical solutions. On the other hand, the latest theoretical analysis [24,25,28] implies that the penalty factor affects the convergence order of the numerical solutions. According to Theorem 1, an option is $\alpha =C_{s}h^{-1}$ for linear basis function, where $C_{s}$ is a constant related to the problem to be solved. Figure 5 shows the log-log plots of the errors ${∥u-u_{h}∥}_{H^{1}\left(\Omega \right)}$ and ${∥p-p_{h}∥}_{L^{2}\left(\Omega \right)}$ with respect to the nodal spacing *h* for different $C_{s}$. Clearly, $C_{s}$ affects the accuracy of the numerical solutions, but hardly impacts the convergence order. By comparison, a great choice is $\alpha =1000h^{-1}$ from a precision point of view, and the error bounds have been tabulated in Table 1. It is clear that the numerical convergence orders are consistent with the theoretical analysis.

**Figure 6:**
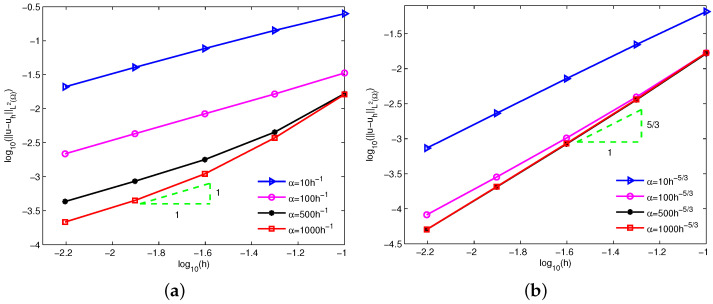
Errors of ${∥u-u_{h}∥}_{L^{2}\left(\Omega \right)}$ based on (**a**) $\alpha =C_{s}h^{-1}$ and (**b**) $\alpha =C_{s}h^{-5/3}$ for example 1.

Moreover, it can be known from Theorem 2 that a valid choice is $\alpha =C_{s}h^{-5/3}$ for the $L^{2}$ norm of velocity errors in terms of linear basis function. Figure 6 displays the log-log plots of the errors ${∥u-u_{h}∥}_{L^{2}\left(\Omega \right)}$ with respect to the nodal spacing *h* for $\alpha =C_{s}h^{-1}$ and $\alpha =C_{s}h^{-5/3}$. Similarly, $\alpha =1000h^{-5/3}$ is an excellent option. Meanwhile, the numerical errors of $\alpha =1000h^{-1}$ and $\alpha =1000h^{-5/3}$ have been shown in Table 2. Visibly, the numerical convergence order of velocity is 1/3 order higher than the theoretical result in terms of $L^{2}$ norm, but the numerical errors of $\alpha =1000h^{-1}$ still accord with the theoretical analysis.

### 7.2. Example 2

The second example considers Stokes problem (1) with ν=0.1. The exact solutions are

$$u_{1}=2\pi \sin^{2}\left(\pi x_{1}\right)\sin\left(\pi x_{2}\right)\cos\left(\pi x_{2}\right),$$

$$u_{2}=-2\pi \sin\left(\pi x_{1}\right)\cos\left(\pi x_{1}\right)\sin^{2}\left(\pi x_{2}\right),$$

$$p=\cos\left(\pi x_{1}\right)\cos\left(\pi x_{2}\right).$$

**Figure 7:**
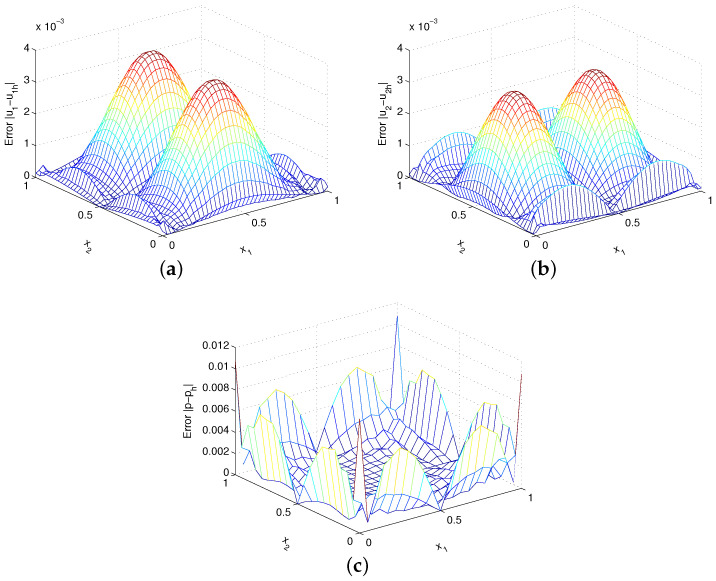
Plots of (**a**) errors $|u_{1}-u_{1h}|$, (**b**) errors $|u_{2}-u_{2h}|$ and (**c**) errors $|p-p_{h}|$ for example 2.

Figure 7 shows the absolute errors $|u_{1}-u_{1h}|$, $|u_{2}-u_{2h}|$ and $|p-p_{h}|$ with $\alpha =1000h^{-1}$. The uniform 41×41 velocity nodes are distributed and a linear basis is adopted in these numerical solutions. Evidently, the method developed in this paper obtains very accurate numerical results. The numerical errors have been tabulated in Table 3 and the results of $\alpha =1000h^{-5/3}$ have also been contained. Clearly, the optimal numerical convergence rate of velocity can reach the second order in $L^{2}$ norm for $\alpha =1000h^{-5/3}$, which is 1/3 order higher than the theoretical suboptimal convergence result. However, the numerical convergence orders for both velocity and pressure are consistent with the theoretical analysis for $\alpha =1000h^{-1}$.

### 7.3. Example 3

**Figure 8:**
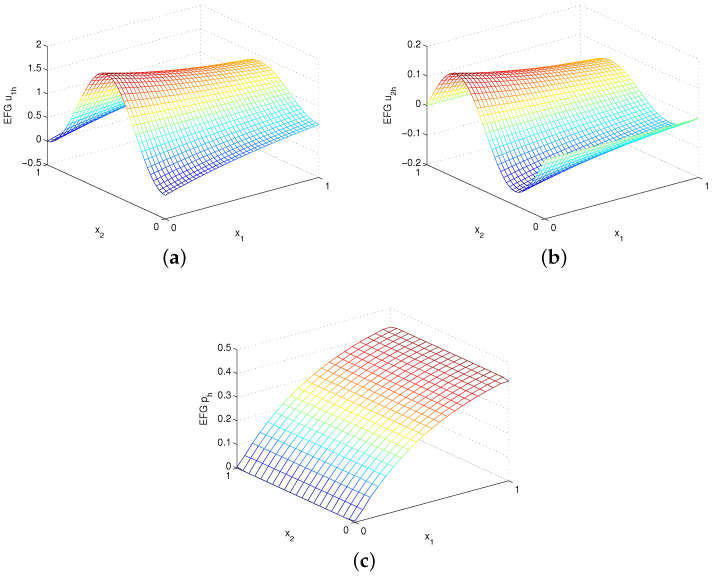
Plots of (**a**) EFG $u_{1h}$, (**b**) EFG $u_{2h}$ and (**c**) EFG $p_{h}$ with Re=40 for example 3.

**Figure 9:**
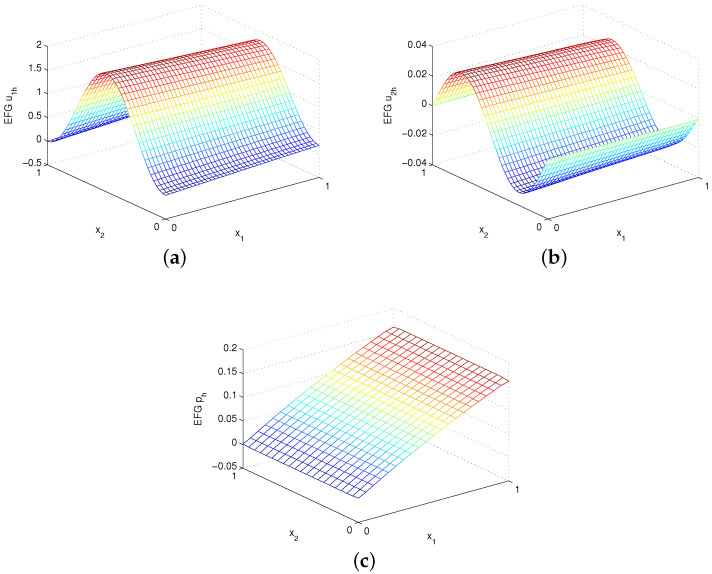
Plots of (**a**) EFG $u_{1h}$, (**b**) EFG $u_{2h}$ and (**c**) EFG $p_{h}$ with Re=200 for example 3.

The third example considers Kovasznay flow [6]. The exact solutions are:

$$u_{1}=1-e^{\lambda x_{1}}\cos\left(2\pi x_{2}\right),$$

$$u_{2}=\frac{\lambda }{2\pi }e^{\lambda x_{1}}\sin\left(2\pi x_{2}\right),$$

$$p=\frac{1}{2}\left(1-e^{2\lambda x_{1}}\right),$$

where $\lambda =\frac{Re}{2}-{\left(4\pi^{2}+\frac{Re^{2}}{4}\right)}^{1/2}$ and $Re=\frac{1}{\nu }$. The EFG numerical solutions $u_{1h}$, $u_{2h}$ and $p_{h}$ are shown in Figure 8 for Re=40 and Figure 9 for Re=200 using uniform 41×41 velocity nodes and $\alpha =1000h^{-1}$. Again, the EFG method gains very accurate numerical solutions. Table 4 and Table 5 give the errors for Re=40 and Re=200, respectively. Obviously, except that the numerical convergence order of velocity of $\alpha =1000h^{-5/3}$ is second order in $L^{2}$ norm, the numerical convergence orders are still in good agreement with the theoretical analysis for $\alpha =1000h^{-1}$.

### 7.4. Example 4

**Figure 10:**
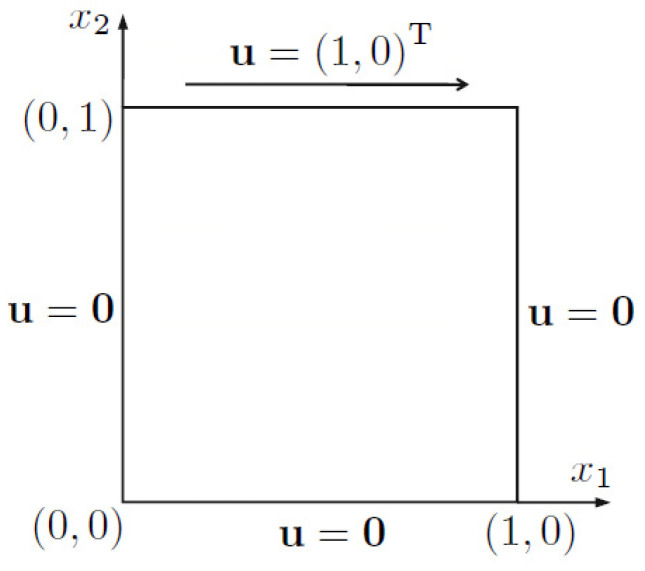
Schematic diagram of example 4.

The last example considers the lid-driven cavity flow problem, which is often regarded as a typical benchmark for incompressible flows. The body force f=0. Figure 10 shows boundary conditions. On the top side $u={\left(1,0\right)}^{T}$ is given, and other sides are no-slip.

**Figure 11:**
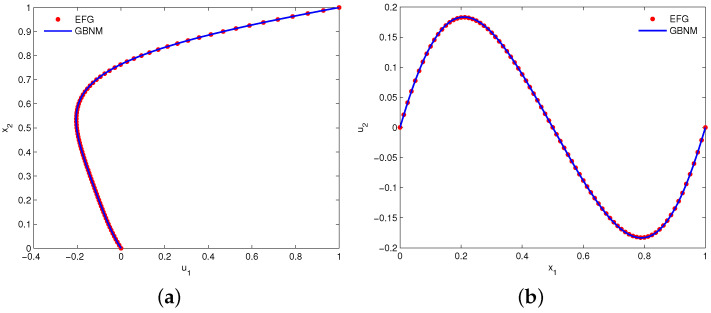
Plots of (**a**) $u_{1}$ along the vertical centerline $x_{1}=0.5$ and (**b**) $u_{2}$ along the horizontal centerline $x_{2}=0.5$ for example 4.

**Figure 12:**
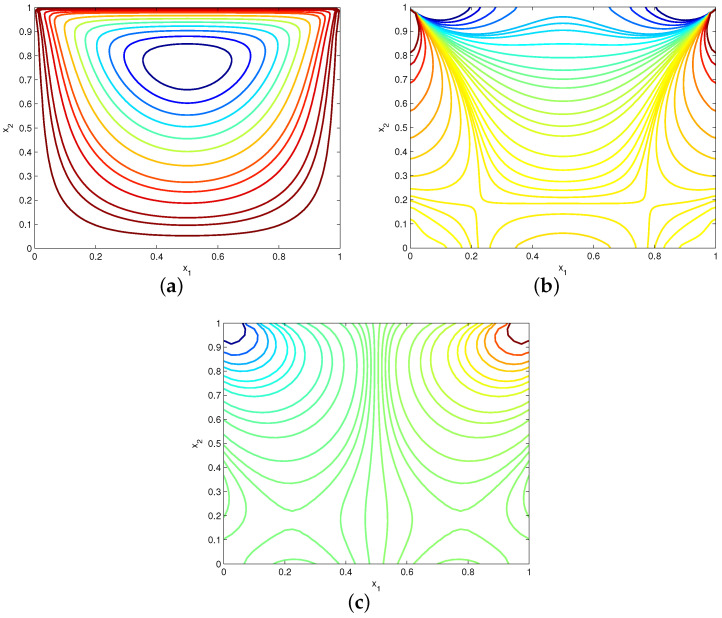
Plots of (**a**) streamline, (**b**) vorticity contour and (**c**) pressure contour for example 4.

Figure 11 shows the EFG solutions of velocities $u_{1}$ and $u_{2}$ along the vertical centerline $x_{1}=0.5$ and horizontal centerline $x_{2}=0.5$, respectively. The numerical results are derived by using uniform 81×81 velocity nodes and $\alpha =1000h^{-1}$. Meanwhile, the results of the Galerkin boundary node method (GBNM) [11] are also plotted in this figure for comparison. Clearly, the EFG solutions are in good agreement with the GBNM results. Moreover, Figure 12 displays the computed plots of streamline, vorticity contour and pressure contour. It can be found that stable numerical results of velocity and pressure are achieved.

## 8. Conclusions

In this paper, we presented and analyzed a penalty-based EFG method for Stokes problems. The penalty method allows the use of the MLS approximation to generate trial and test functions in the modified weak form. A priori errors of the penalty method are determined on the Dirichlet boundary and in the problem domain respectively, which state the feasibility of the penalty method in a continuous sense. For the penalized Stokes problems, the existence and uniqueness of the weak solution are proved under a rational assumption, which provides a valid foundation for the EFG numerical discretization. Under the condition of discrete inf-sup, error estimates with a penalty factor are provided in $H^{1}$ and $L^{2}$ norms for velocity approximation and in $L^{2}$ norm for pressure approximation.

Numerical results reveal that the proposed method exhibits good numerical accuracy and agrees well with the theoretical prediction. Note that for linear basis functions, the numerical convergence order of velocity can reach the second order in $L^{2}$ norm, but we have only theoretically obtained a suboptimal convergence order of velocity. Therefore, more research is required on the theoretical analysis of the present method for deriving the optimal convergence order of velocity in $L^{2}$ norm. In addition, how to reduce the condition numbers is an important research topic.

## Figures and Tables

**Table 1:** Errors and convergence orders using $\alpha =1000h^{-1}$ for example 1.

*h*	${∥u-u_{h}∥}_{H^{1}\left(\Omega \right)}$	Order	${∥p-p_{h}∥}_{L^{2}\left(\Omega \right)}$	Order
1/10	$7.2382\times 10^{-1}$		$1.0732\times 10^{-1}$	
1/20	$3.5547\times 10^{-1}$	1.03	$4.4393\times 10^{-2}$	1.27
1/40	$1.7753\times 10^{-1}$	1.00	$2.1408\times 10^{-2}$	1.05
1/80	$8.8876\times 10^{-2}$	1.00	$1.0994\times 10^{-2}$	0.96

**Table 2:** Errors and convergence orders of velocity for example 1.

*h*	$\alpha =1000h^{-1}$		$\alpha =1000h^{-5/3}$	
	${∥u-u_{h}∥}_{L^{2}\left(\Omega \right)}$	Order	${∥u-u_{h}∥}_{L^{2}\left(\Omega \right)}$	Order
1/10	$1.6257\times 10^{-2}$		$1.6877\times 10^{-2}$	
1/20	$3.7226\times 10^{-3}$	2.12	$3.6534\times 10^{-3}$	2.21
1/40	$1.1000\times 10^{-3}$	1.75	$8.5527\times 10^{-4}$	2.09
1/80	$4.4776\times 10^{-4}$	1.30	$2.0661\times 10^{-4}$	2.05
1/160	$2.1579\times 10^{-4}$	1.05	$5.0659\times 10^{-5}$	2.03

**Table 3:** Errors and convergence orders for example 2.

*h*	$\alpha =1000h^{-1}$		$\alpha =1000h^{-5/3}$		$\alpha =1000h^{-1}$			
	${∥u-u_{h}∥}_{L^{2}\left(\Omega \right)}$	Order	${∥u-u_{h}∥}_{L^{2}\left(\Omega \right)}$	Order	${∥u-u_{h}∥}_{H^{1}\left(\Omega \right)}$	Order	${∥p-p_{h}∥}_{L^{2}\left(\Omega \right)}$	Order
1/10	$1.9490\times 10^{-2}$		$1.8831\times 10^{-2}$		1.1118		$1.7577\times 10^{-2}$	
1/20	$6.8658\times 10^{-3}$	1.51	$4.6056\times 10^{-3}$	2.03	$5.6761\times 10^{-1}$	0.97	$9.1652\times 10^{-3}$	0.94
1/40	$3.2729\times 10^{-3}$	1.07	$1.1270\times 10^{-3}$	2.03	$2.8562\times 10^{-1}$	0.99	$5.0062\times 10^{-3}$	0.87
1/80	$1.6865\times 10^{-3}$	0.96	$2.7761\times 10^{-4}$	2.02	$1.4315\times 10^{-1}$	0.99	$2.7330\times 10^{-3}$	0.87

**Table 4:** Errors and convergence orders with Re=40 for example 3.

*h*	$\alpha =1000h^{-1}$		$\alpha =1000h^{-5/3}$		$\alpha =1000h^{-1}$			
	${∥u-u_{h}∥}_{L^{2}\left(\Omega \right)}$	Order	${∥u-u_{h}∥}_{L^{2}\left(\Omega \right)}$	Order	${∥u-u_{h}∥}_{H^{1}\left(\Omega \right)}$	Order	${∥p-p_{h}∥}_{L^{2}\left(\Omega \right)}$	Order
1/10	$4.5081\times 10^{-3}$		$8.0507\times 10^{-3}$		$2.4316\times 10^{-1}$		$2.3505\times 10^{-3}$	
1/20	$1.0919\times 10^{-3}$	2.04	$1.5885\times 10^{-3}$	2.34	$1.2151\times 10^{-1}$	1.00	$5.9778\times 10^{-4}$	1.97
1/40	$3.8417\times 10^{-4}$	1.51	$3.0895\times 10^{-4}$	2.36	$6.0934\times 10^{-2}$	0.99	$1.9001\times 10^{-4}$	1.65
1/80	$1.7917\times 10^{-4}$	1.01	$6.4406\times 10^{-5}$	2.26	$3.0530\times 10^{-2}$	0.97	$8.0235\times 10^{-5}$	1.24

**Table 5:** Errors and convergence orders with Re=200 for example 3.

*h*	$\alpha =1000h^{-1}$		$\alpha =1000h^{-5/3}$		$\alpha =1000h^{-1}$			
	${∥u-u_{h}∥}_{L^{2}\left(\Omega \right)}$	Order	${∥u-u_{h}∥}_{L^{2}\left(\Omega \right)}$	Order	${∥u-u_{h}∥}_{H^{1}\left(\Omega \right)}$	Order	${∥p-p_{h}∥}_{L^{2}\left(\Omega \right)}$	Order
1/10	$6.2224\times 10^{-3}$		$1.0417\times 10^{-2}$		$3.2721\times 10^{-1}$		$5.8460\times 10^{-4}$	
1/20	$1.6929\times 10^{-3}$	1.87	$2.0728\times 10^{-3}$	2.32	$1.6389\times 10^{-1}$	0.99	$1.6378\times 10^{-4}$	1.83
1/40	$6.8296\times 10^{-4}$	1.31	$4.0807\times 10^{-4}$	2.34	$8.2268\times 10^{-2}$	0.99	$6.2079\times 10^{-5}$	1.39
1/80	$3.3516\times 10^{-4}$	1.03	$8.6847\times 10^{-5}$	2.23	$4.1244\times 10^{-2}$	0.99	$2.9734\times 10^{-5}$	1.06

## Data Availability

Not applicable.

## References

[1] Babuška I., Banerjee U., Osborn J.E. (2003). Survey of meshless and generalized finite element methods: A unified approach. Acta Numer..

[2] Li S.H., Liu W.K. (2004). Meshfree Particle Methods.

[3] Liu G.R. (2009). Meshfree Methods: Moving beyond the Finite Element Method.

[4] Cheng Y.M. (2015). Meshless Methods.

[5] Sun T., Li J.W., Zhao J.P., Feng X.L. (2019). Least-squares RBF-FD method for the incompressible Stokes equations with the singular source. Numer. Heat Transf. Part A Appl..

[6] Park S.K., Jo G., Choe H.J. (2016). Existence and stability in the virtual interpolation point method for the Stokes equations. J. Comput. Phys..

[7] Song L.N., Li P.W., Gu Y., Fan C.M. (2020). Generalized finite difference method for solving stationary 2D and 3D Stokes equations with a mixed boundary condition. Comput. Math. Appl..

[8] Li J.W., Gao Z.M., Dai Z.H., Feng X.L. (2020). Divergence-free radial kernel for surface Stokes equations based on the surface helmholtz decomposition. Comput. Phys. Commun..

[9] Choe H.J., Kim D.W., Kim H.H., Kim Y. (2001). Meshless method for the stationary incompressible Navier-Stokes equations. Discret. Contin. Dyn. Syst. B..

[10] Kumar V.V.K.S., Kumar B.V.R., Das P.C. (2006). Weighted extended B-spline method for the approximation of the stationary Stokes problem. J. Comput. Appl. Math..

[11] Li X.L., Zhu J.L. (2009). A meshless Galerkin method for Stokes problems using boundary integral equations. Comput. Methods Appl. Mech. Eng..

[12] Najafi M., Dehghan M., Šarler B., Kosec G., Mavrič B. (2022). Divergence-free meshless local Petrov-Galerkin method for Stokes flow. Eng. Comput..

[13] Belytschko T., Lu Y.Y., Gu L. (1994). Element-free Galerkin methods. Int. J. Numer. Methods Eng..

[14] Lancaster P., Salkauskas K. (1981). Surfaces generated by moving least squares methods. Math. Comput..

[15] Cheng H., Peng M.J., Cheng Y.M. (2018). The dimension splitting and improved complex variable element-free Galerkin method for 3-dimensional transient heat conduction problems. Int. J. Numer. Methods Eng..

[16] Wan J.S., Li X.L. (2022). Analysis of a superconvergent recursive moving least squares approximation. Appl. Math. Lett..

[17] Li X.L. (2022). Theoretical analysis of the reproducing kernel gradient smoothing integration technique in Galerkin meshless methods. J. Comput. Math..

[18] Yu S.Y., Peng M.J., Cheng H., Cheng Y.M. (2019). The improved element-free Galerkin method for three-dimensional elastoplasticity problems. Eng. Anal. Bound. Elem..

[19] Zheng Z.Y., Li X.L. (2022). Theoretical analysis of the generalized finite difference method. Comput. Math. Appl..

[20] Wang J.F., Sun F.X., Cheng Y.M. (2012). An improved interpolating element-free Galerkin method with a nonsingular weight function for two-dimensional potential problems. Chin. Phys. B.

[21] Sun F.X., Wang J.F., Cheng Y.M., Huang A.X. (2015). Error estimates for the interpolating moving least-squares method in *n*-dimensional space. Appl. Numer. Math..

[22] Li X.L., Li S.L. (2021). A fast element-free Galerkin method for the fractional diffusion-wave equation. Appl. Math. Lett..

[23] Wu J.C., Wang D.D. (2021). An accuracy analysis of Galerkin meshfree methods accounting for numerical integration. Comput. Methods Appl. Mech. Eng..

[24] Li X.L., Li S.L. (2016). On the stability of the moving least squares approximation and the element-free Galerkin method. Comput. Math. Appl..

[25] Zhang T., Li X.L. (2020). Analysis of the element-free Galerkin method with penalty for general second-order elliptic problems. Appl. Math. Comput..

[26] Zhang J.P., Shen Y., Hu H.Y., Gong S.G., Wu S.Y., Wang Z.Q., Huang J. (2021). Transient heat transfer analysis of orthotropic materials considering phase change process based on element-free Galerkin method. Int. Commun. Heat Mass..

[27] Ding R., Shen Q., Yao Y. (2022). The element-free Galerkin method for the dynamic Signorini contact problems with friction in elastic materials. Appl. Math. Comput..

[28] Zhang T., Li X.L., Xu L.W. (2022). Error analysis of an implicit Galerkin meshfree scheme for general second-order parabolic problems. Appl. Numer. Math..

[29] Li J., Chen Z.X. (2008). A new local stabilized nonconforming finite element method for the Stokes equations. Computing.

[30] Evans L.C. (2010). Partial Differential Equations.

[31] Babuška I. (1973). The finite element method with penalty. Math. Comput..

